# Pharmacological Activation of Sirt1 Ameliorates Polyglutamine-Induced Toxicity through the Regulation of Autophagy

**DOI:** 10.1371/journal.pone.0064953

**Published:** 2013-06-10

**Authors:** Bae Hyun Shin, Yunki Lim, Hye Jin Oh, Sang Min Park, Sun-Kyung Lee, Joohong Ahnn, Do Han Kim, Woo Keun Song, Tae Hwan Kwak, Woo Jin Park

**Affiliations:** 1 Global Research Laboratory, Gwangju Institute of Science and Technology (GIST), Gwangju, Korea; 2 College of Life Sciences, Gwangju Institute of Science and Technology (GIST), Gwangju, Korea; 3 Department of Life Science, Hanyang University, Seoul, Korea; 4 The Research Institute for Natural Sciences, Hanyang University, Seoul, Korea; 5 R&D Institute, Mazence Inc., Suwon, Korea; University Medical Center Groningen, The Netherlands

## Abstract

Intracellular accumulation of polyglutamine (polyQ)-expanded Huntingtin (Htt) protein is a hallmark of Huntington’s disease (HD). This study evaluated whether activation of Sirt1 by the anti-cancer agent, β-lapachone (β-lap), induces autophagy in human neuroblastoma SH-SY5Y cells, thereby reducing intracellular levels of polyQ aggregates and their concomitant cytotoxicity. Treatment of cells with β-lap markedly diminished the cytotoxicity induced by forced expression of Htt exon 1 containing a pathogenic polyQ stretch fused to green fluorescent protein (HttEx1(97Q)-GFP). β-lap increased autophagy in SH-SY5Y cells, as evidenced by the increased formation of LC3-II and autolysosomes. Furthermore, β-lap reduced HttEx1(97Q)-GFP aggregation, which was significantly prevented by co-incubation with 3-methyladenine, an inhibitor of autophagy. β-lap increased Sirt1 activity, as shown by the increased deacetylation of the Sirt1 substrates, PARP-1 and Atg5, and the nuclear translocation of FOXO1. Both the induction of autophagy and attenuation of HttEx1(97Q)-GFP aggregation by β-lap were significantly prevented by co-incubation with sirtinol, a general sirtuin inhibitor or by co-transfection with shRNA against Sirt1. The pro-autophagic actions of β-lap were further investigated in a transgenic *Caenorhabditis elegans* (*C. elegans*) line that expressed Q67 fused to cyanine fluorescent protein (Q67). Notably, β-lap reduced the number of Q67 puncta and restored Q67-induced defects in motility, which were largely prevented by pre-treatment with RNAi against sir-2.1, the *C. elegans* orthologue of Sirt1. Collectively, these data suggest that β-lap induces autophagy through activation of Sirt1, which in turn leads to a reduction in polyQ aggregation and cellular toxicity. Thus, β-lap provides a novel therapeutic opportunity for the treatment of HD.

## Introduction

Huntington’s disease (HD) is an autosomal dominant neurodegenerative disorder caused by a CAG repeat expansion, which is translated into a long polyglutamine (polyQ) tract [1]. The gene involved in HD encodes a 350-kDa protein termed Huntingtin (Htt), which contains a highly polymorphic CAG repeat in exon 1. The number of CAG copies ranges from 10 to 35 in unaffected individuals, but exceeds 36 in patients with HD. The aggregation of mutant Htt containing the expanded polyQ tract is a hallmark of HD [2], [3]. Other neurodegenerative disorders (i.e., spinocerebellar ataxias) are similarly caused by the aggregation of proteins containing pathogenic polyQ stretches. The toxicity of polyQ aggregates is attributed to an impaired ubiquitin-proteasome system [4]. Thus, elimination of polyQ aggregates is an attractive therapeutic strategy for the treatment of neurodegenerative disorders including HD.

Autophagy is the process of bulk degradation of cytoplasmic proteins or organelles in the lytic compartment. This process involves the formation of autophagosomes, double-membraned vesicular structures that non-specifically sequester portions of the cytoplasm and ultimately fuse with protease-containing lysosomes [5]. Autophagy eliminates toxic polyQ aggregates and reduces their cytotoxicity [6], [7]. Compromised autophagic activity in HD is mainly caused by the defective recognition of cytosolic cargoes by autophagic vacoules, and the inefficient engulfment of cytosolic components by the autophagosome contributes to the cellular toxicity associated with HD [8]. Therefore, activation of autophagy can be an effective therapeutic modality for the treatment of HD and other polyQ diseases.

Sirt1, the mammalian ortholog of yeast silent information regulator (Sir2), is a nicotinamide adenine dinucleotide (NAD^+^)-dependent class III histone deacetylase that is associated with the regulation of lifespan, metabolism, and cellular survival [9], [10], [11]. Recently, Sirt1 was shown to induce autophagy by deacetylating key autophagy-related proteins, including Atg5, Atg7, and Atg8 [12]. This finding led us to hypothesize that increased Sirt1 activity may accelerate the autophagy-mediated elimination of polyQ aggregates, thereby ameliorating their harmful actions.

β-lapachone (β-lap), a natural *o*-naphthoquinone compound, is a substrate of NADH:quinone oxidoreductase (NQO1). NQO1 mediates the reduction of β-lap by using NADH as an electron source [13]. Reduced β-lap is unstable and rapidly re-oxidized. This futile β-lap redox cycle is coupled with the oxidation of NADH to NAD^+^. Because Sirt1 activity strictly requires NAD^+^ as a cofactor, β-lap may increase Sirt1 activity by increasing cellular NAD^+^ levels [14], [15], [16]. Therefore, we investigated whether β-lap can increase the clearance of polyQ aggregates and reduce polyQ-mediated cytotoxicity through Sirt1-dependent induction of autophagy in SH-SY5Y cells.

## Results

### β-lap Prevents polyQ-mediated Cytotoxicity

**Figure 1:**
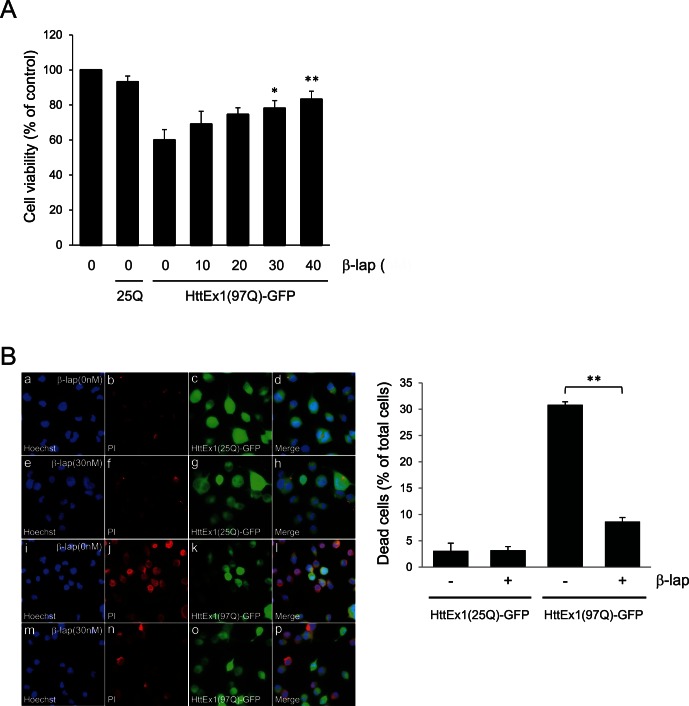
β-lap prevents polyQ-mediated cytotoxicity. **A.** Human neuroblastoma SH-SY5Y cells were transfected with pcDNA, pcDNA-HttEx1(25Q)-GFP, or the (97Q)-GFP plasmid. After incubation for 24 h, cells were treated with various concentrations of β-lap for 12 h. Cell viability was determined by using the MTT assay. Each bar and error bar represents the mean ± SD (n = 3); *p<0.05, **p<0.01 vs. no β-lap treatment. **B.** SH-SY5Y cells were transfected with pcDNA-HttEx1(25Q) or the (97Q)-GFP plasmid. After incubation for 24 h, cells were treated with DMSO or 30 nM β-lap for 12 h and exposed to 200 µM H_2_O_2_ for an additional 5 h. Cells were co-stained with Hoechst 33342 or PI. Cells stained by PI represent dead cells, whereas Hoechst 33342 staining reveals all nuclei. Percentage cell death was calculated by determining the ratio of PI-stained cells to Hoechst-stained cells. Each bar and error bar represents the mean ± SD (n = 4); **p<0.01.

To assess polyQ aggregation and polyQ-mediated cytotoxicity, we generated two plasmid constructs that encode fusion proteins comprising Htt exon 1 (containing 25 or 97 glutamine stretches) and green fluorescent protein (GFP), referred to as HttEx1(25Q)-GFP and HttEx1(97Q)-GFP, respectively. Expression of HttEx1(97Q)-GFP but not HttEx1(25Q)-GFP in human neuroblastoma SH-SY5Y cells led to markedly reduced cell viability (Fig. 1A). Co-treatment with β-lap for 12 h significantly ameliorated the cytotoxicity of HttEx1(97Q)-GFP in a dose-dependent manner (Fig. 1A). Maximum effects were seen with 30–40 nM β-lap. Thus, we used 30 nM β-lap for further investigations.

Cells expressing HttEx1-GFP fusion proteins containing a pathogenic polyQ stretch are highly sensitive to oxidative stress [17]. Consistent with this, exposure of HttEx1(97Q)-GFP-expressing SH-SY5Y cells to 200 µM hydrogen peroxide (H_2_O_2_) yielded a substantial number of propidium iodide (PI)-stained dead cells, whereas cells expressing HttEx1(25Q)-GFP were relatively insensitive. Pre-treatment with β-lap significantly prevented the HttEx1(97Q)-GFP-mediated hypersensitivity of SH-SY5Y cells to oxidative stress (Fig. 1B). These data indicate that β-lap precluded polyQ-mediated cell death.

### β-lap Reduces the Formation of polyQ Aggregates by Inducing Autophagy

**Figure 2:**
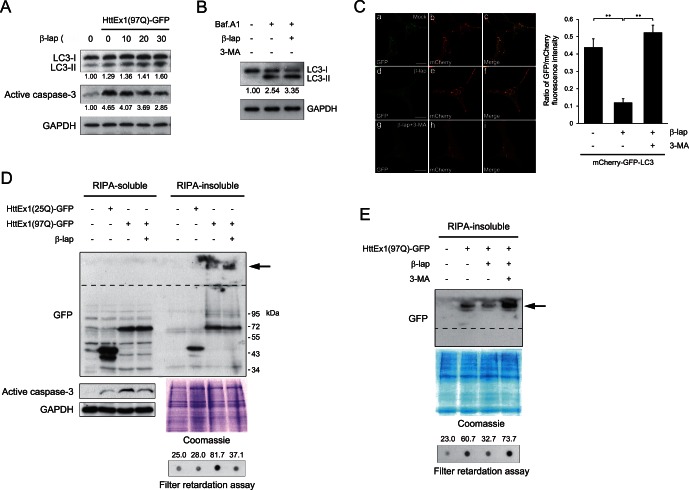
β-lap reduces polyQ aggregation through the induction of autophagy. **A.** SH-SY5Y cells were transfected with the pcDNA or pcDNA-HttEx1(97Q)-GFP plasmid. After incubation for 24 h, cells were treated with various concentrations of β-lap for 12 h. Cell lysates were separated by SDS-PAGE, transferred onto a PVDF membrane, and probed with an antibody against LC3 or active caspase-3. GAPDH (detected with an anti-GAPDH antibody) was used as the loading control. The intensity of the protein bands was determined by using NIH ImageJ software. Numbers shown represent the expression ratios of LC3-II to LC-I, or the expression levels of active caspase-3. **B.** SH-SY5Y cells were treated with 30 nM β-lap for 7 h and further incubated with 400 nM bafilomycin A_1_ for 5 h. Cell lysates were separated by SDS-PAGE, transferred onto a PVDF membrane, and probed with an antibody against LC3. The intensity of the protein bands was determined by using NIH ImageJ software. Numbers shown represent the expression ratios of LC3-II to LC-I. **C.** SH-SY5Y cells were transfected with a mCherry-GFP-LC3 plasmid. After incubation for 24 h, cells were treated with 30 nM β-lap in the absence or presence of 10 mM 3-MA for 12 h. Cells were then fixed and observed under a confocal microscope. The ratios of GFP/mCherry fluorescence were plotted. Each bar and error bar represents the mean ± SD (n = 12); **p<0.01. **D.** SH-SY5Y cells were transfected with pcDNA-HttEx1(25Q) or (97Q)-GFP plasmid. After incubation for 24 h, cells were treated with DMSO or 30 nM β-lap for 12 h. Cell lysates were separated into RIPA-soluble and RIPA-insoluble fractions and analysed by Western blotting with an antibody against GFP (a–d). (a) PolyQ aggregates trapped in the stacking gel are indicated by an arrow, and the boundary between the stacking and separating gels is shown by a broken line. (b) Changes in the expression levels of active caspase-3 are shown to verify the activity of β-lap. GAPDH (detected with an anti-GAPDH antibody (c) and Coomassie blue staining of gels (d) were performed to ensure equal protein loading. (e) The accumulation of insoluble HttEx1(97Q) protein was examined by a filter retardation assay. The intensity of the spots was determined using NIH ImageJ software after immunoreaction with an antibody against GFP. Numbers shown are arbitrary indicators of spot intensity. **E.** SH-SY5Y cells were transfected with pcDNA or pcDNA-HttEx1(97Q)-GFP plasmid. After incubation with plasmids for 24 h, cells were incubated for another 36 h in the absence or presence of 30 nM β-lap and 10 mM 3-MA. RIPA-insoluble fractions were analysed as in (D).

We next investigated whether β-lap regulates autophagy. Western blot analysis revealed that β-lap increased autophagosome formation in HttEx1(97Q)-GFP-expressing SH-SY5Y cells in a dose-dependent manner, as shown by the increased conversion of LC3-I, the cytosolic precursor form of autophagy modifier protein LC3, to LC3-II, the membrane-bound processed form (Fig. 2A). β-lap significantly prevented HttEx1(97Q)-GFP-mediated activation of caspase-3, an “executioner” protease that directly regulates apoptosis (Fig. 2A). Inhibition of autophagosome degradation by a saturating concentration of bafilomycin A_1_ resulted in an increase in the LC3-II level. The LC3-II level was further increased by co-treatment with β-lap, indicating that β-lap acted by increasing autophagosome formation, not by impairing autophagosome-lysosome fusion (Fig. 2B). We utilized a mCherry-GFP-LC3 reporter construct to monitor the progression of autophagy. A decrease in the ratio of the fluorescence of acid-sensitive GFP to that of acid-insensitive mCherry indicates increased autophagosome-lysosome fusion (autolysosome formation). The ratio of GFP fluorescence to mCherry fluorescence was significantly reduced by β-lap treatment, indicating that autolysosome formation was increased by β-lap. The pro-autophagic activity of β-lap was completely abrogated by 3-methyladenine (3-MA), an inhibitor of autophagy (Fig. 2C). These data support the hypothesis that β-lap prevents polyQ-mediated cytotoxicity by inducing autophagy.

Extracts of SH-SY5Y cells expressing HttEx1(25Q)-GFP or HttEx1(97Q)-GFP were fractionated into RIPA-soluble and -insoluble fractions and then subjected to Western blotting. As expected, HttEx1(25Q)-GFP (∼50 kDa) was present mostly in the soluble fraction, whereas HttEx1(97Q)-GFP (∼70 kDa) was found in both soluble and insoluble fractions. The indistinct protein band in the RIPA-insoluble fraction (arrow) represented insoluble aggregates of HttEx1(97Q)-GFP trapped in the stacking gel; these aggregates were significantly reduced by treatment with β-lap (Fig. 2D, top). The RIPA-insoluble fraction was also subjected to a filter retardation assay, in which the aggregated proteins are trapped in the filter. Substantial amounts of HttEx1(97Q)-GFP were retained by the filter in this assay; however, β-lap again significantly attenuated aggregation of the fusion protein (Fig. 2D, bottom). Importantly, the ability of β-lap to prevent the formation of polyQ aggregates was completely abolished in the presence of 3-MA, as assessed by both Western blotting and filter retardation assays (Fig. 2E). Collectively, these results indicate that β-lap eliminated polyQ aggregation by inducing autophagy.

### β-lap Reduces polyQ Aggregates through Activation of Sirt1

**Figure 3:**
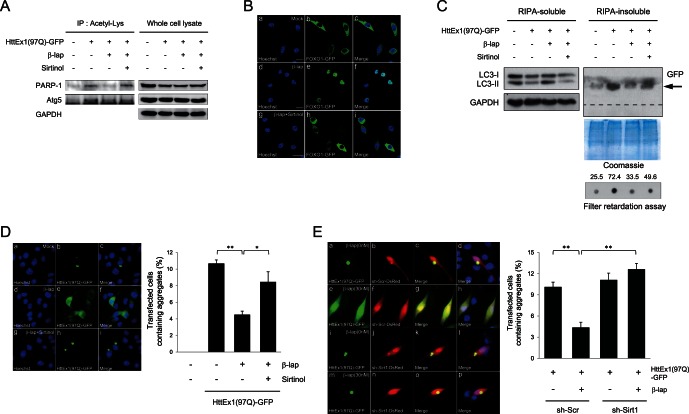
β-lap reduces polyQ aggregation through the activation of Sirt1. **A.** SH-SY5Y cells were transfected with pcDNA or the pcDNA-HttEx1(97Q)-GFP plasmid. After incubation for 24 h, the cells were treated with β-lap in the absence or presence of sirtinol. Cells were then lysed and immunoprecipitated with anti-acetyl lysine antibody. The precipitates were analyzed by Western blot analysis with anti-PARP-1 or anti-Atg5 antibodies. **B.** SH-SY5Y cells were transfected with a FOXO1-GFP plasmid and treated with β-lap in the absence or presence of sirtinol. The cells were then fixed and observed under a confocal microscope. **C.** SH-SY5Y cells were transfected with pcDNA or pcDNA-HttEx1(97Q)-GFP plasmid. After incubation with plasmids for 24 h, cells were incubated for another 12 h in the presence of absence of 30 nM β-lap and 50 µM sirtinol. Cell lysates were separated into RIPA-soluble and RIPA-insoluble fractions and analysed by Western blotting with an anti-LC3 or anti-GFP antibodies. PolyQ aggregates trapped in the stacking gel are indicated by an arrow, and the boundary between stacking and separating gels is shown by a broken line. GAPDH blotting and Coomassie staining were performed to ensure equal protein loading. The accumulation of insoluble HttEx1(97Q) protein was examined in a filter retardation assay. The intensity of the spots was determined using NIH ImageJ software after immunoreaction with an anti-GFP antibody. Numbers shown are arbitrary indicators of spot intensity. **D.** SH-SY5Y cells were transfected with pcDNA-HttEx1(97Q) and examined for the polyQ aggregate-clearing ability of β-lap. After incubation with β-lap or β-lap plus sirtinol, the cells were fixed, stained with Hoechst 33342, and observed under a fluorescence microscope. The percentage of transfected cells containing GFP-labelled aggregates was plotted. Each bar and error bar represents the mean ± SD (n = 4); *p<0.05, **p<0.01 (Student’s *t*-test). **E.** SH-SY5Y cells were co-transfected with pcDNA-HttEx1(97Q)-GFP and a plasmid harbouring scrambled shRNA (sh-Scr) or Sirt1 shRNA (sh-Sirt1). After incubation with plasmids for 24 h, cells were further incubated in the presence or absence of 30 nM β-lap and observed under a fluorescence microscope. The shRNA-harbouring plasmid expresses a variant of *Discosoma* sp. red fluorescent protein (DsRed); therefore, cells transfected with this plasmid constitutively express DsRed. The percentage of transfected cells containing GFP-labelled aggregates was plotted. Each bar and error bar represents the mean ± SD (n = 8); **p<0.01 (Student’s *t*-test).

We then examined Sirt1 activity in SH-SY5Y cells under various conditions. Acetylated proteins were immunoprecipitated from SH-SY5Y cell extracts using anti-acetyl-lysine antibody and then probed with antibodies to known Sirt1 substrates, poly(ADP-ribose) polymerase-1 (PARP-1) [18] and Atg5 [12]. HttEx1(97Q)-GFP expression resulted in increased acetylation of these proteins, indicating compromised Sirt1 activity. β-lap restored the deacetylation of these proteins, but deacetylation was inhibited by co-treatment with sirtinol, a general sirtuin inhibitor (Fig. 3A). Nuclear translocation of FOXO1 is tightly associated with Sirt1-mediated deacetylation of FOXO1 [19]. We therefore determined the cellular distribution of FOXO1 using a FOXO1-GFP fusion protein in SH-SY5Y cells. While FOXO-GFP localized primarily to the cytoplasm, β-lap treatment resulted in its translocation to the nucleus. Co-treatment with sirtinol completely abolished FOXO1 nuclear translocation (Fig. 3B). These data indicated that β-lap increases Sirt1 activity in SH-SY5Y cells.

The above data and data from other studies [14], [15] show that β-lap activates Sirt1; thus, we tested the hypothesis that autophagy-mediated reduction of polyQ aggregates in response to β-lap requires Sirt1 activity. HttEx1(97Q)-GFP expression reduced the conversion of LC3-I to LC3-II, but β-lap restored the conversion on Western blots. This pro-autophagic activity was abolished by co-treatment with sirtinol (Fig. 3C, left panel). The β-lap-mediated reduction of polyQ aggregation was also significantly inhibited by co-treatment with sirtinol (Fig. 3C, right panel). This finding was further confirmed by fluorescence microscopy. Approximately 10–13% of the HttEx1(97Q)-GFP-expressing SH-SY5Y cells contained protein aggregates, observed as fluorescent puncta. These aggregates were significantly reduced by β-lap, whereas the actions of β-lap were reversed by co-treatment with sirtinol (Fig. 3D).

Next, SH-SY5Y cells were co-transfected with a plasmid encoding HttEx1(97Q)-GFP and a pSIREN-DNR-DsRed-Express plasmid expressing either scrambled shRNA (sh-Scr) or Sirt1 shRNA (sh-Sirt1). Expression of shRNA throughout the cytoplasm and in the nucleus was revealed by the fluorescence of *Discosoma* sp. red fluorescent protein (DsRed; Fig. 3E), while Western blotting showed that transfection of sh-Sirt1 into SH-SY5Y cells reduced the level of Sirt1 by more than 60% (Fig. S1). β-lap effectively reduced the aggregation of HttEx1(97Q)-GFP in cells transfected with sh-Scr. However, β-lap was completely ineffective in cells transfected with sh-Sirt1 (Fig. 3E). Taken together, these data indicate that β-lap induces the autophagic elimination of intracellular polyQ aggregates in SH-SY5Y cells through the activation of Sirt1.

### β-lap Reduces polyQ Aggregates and polyQ-mediated Mobility Dysfunction in *C. elegans*

**Figure 4:**
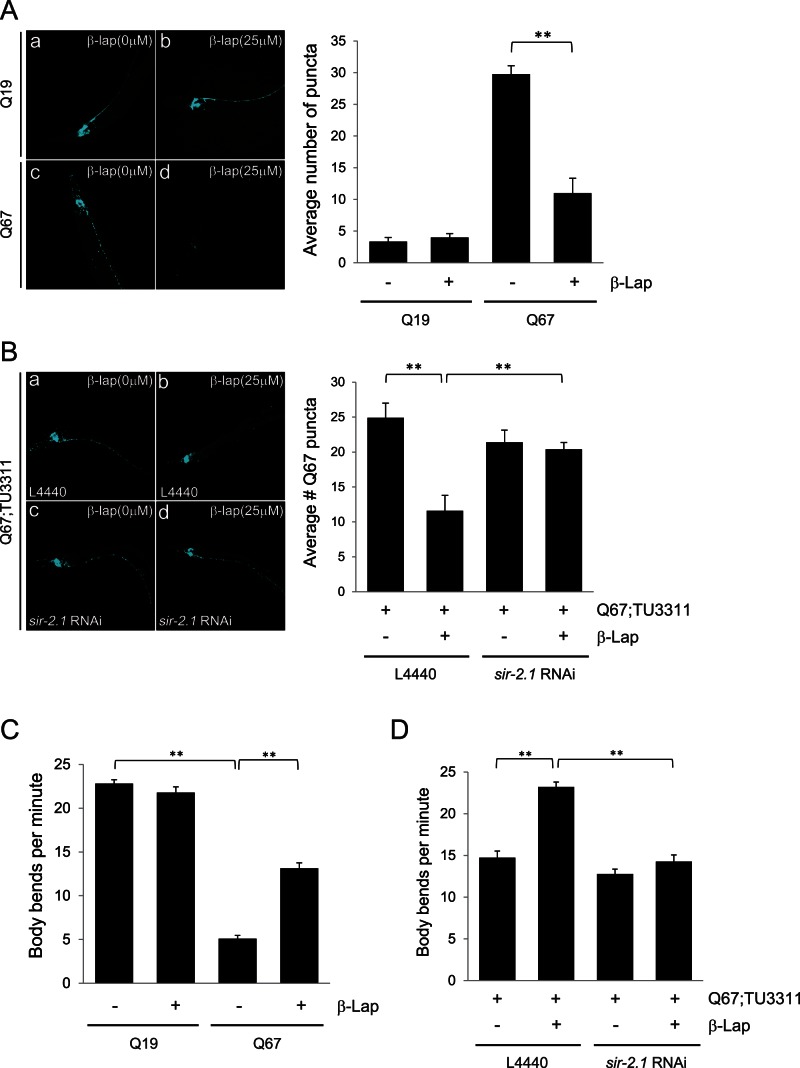
β-lap protects polyQ-expressing neurons from toxicity in *C. elegans*. **A and C.** Fluorescence micrographs of Q19- (a and b) and Q67-expressing *C. elegans* (c and d) are shown in the presence (b and d) and absence (a and c) of 25 µM β-lap. All animals depicted are young adults (4 days post-hatch). The average number of polyQ puncta (A) and body bends during 1 min of continuous movement on agar plates (C) were quantified and plotted. Each bar and error bar represents the mean ± SD (A: n = 10, C: n = 31); **p<0.01 (Student’s *t*-test). **B and D.** RNA interference experiments using *sir-2.1* RNAi in Q67;TU3311 double transgenic worms were performed in the presence (b and d) or absence (a and c) of 25 µM β-lap. The average number of polyQ puncta (B) and body bends during 1 min of continuous movement on agar plates (D) were quantified and plotted. Each bar and error bar represents the mean ± SD (A: n = 10, C: n = 31); **p<0.01 (Student’s *t* test).

Our next goal was to test whether β-lap is capable of suppressing polyQ-induced toxicity *in vivo*. For this purpose, we utilized transgenic *C. elegans* lines expressing cyan fluorescent protein (CFP)-tagged polyQ proteins in neurons under the control of the F25B3.3 promoter [20]. Expression of a polyQ protein containing Q19 fused to CFP (Q19) yields a diffuse neuronal distribution pattern, whereas expression of a polyQ protein containing Q67 fused to CFP (Q67) yields a discrete localization pattern of protein aggregates (Fig. 4A). These results suggest that polyQ length-dependent aggregation occurs in the neurons of *C. elegans*. The number of Q67 aggregates was significantly reduced by treatment with β-lap (Fig. 4A).

To test whether the anti-aggregation effect of β-lap in transgenic worms requires sirtuin activity, we performed RNA interference experiments with RNAi against sir-2.1 (*sir-2.1* RNAi), the *C. elegans* orthologue of Sirt1. Q67-expressing worms were crossed with TU3311 (*Punc-119*::*sid-1*) worms that exhibited an enhanced delivery of double stranded RNA (dsRNA) into neurons. The resulting double transgenic Q67;TU3311 worms were fed with bacteria containing a control plasmid L4440 or a *sir-2.1* RNAi plasmid and simultaneously treated with β-lap (25 µM). While β-lap successfully reduced the Q67 aggregates in worms fed with L4440-containing bacteria, it had no significant effect in worms fed with bacteria containing *sir-2.1* RNAi plasmid (Fig. 4B).

PolyQ aggregate-induced toxicity in neuronal cells is reflected by the reduced locomotive activity of *C. elegans* [21]. The number of body bends per minute was markedly decreased in worms expressing Q67 compared with that in worms expressing Q19. The Q67-mediated locomotion defects were significantly abrogated by β-lap (Fig. 4C). The effect of β-lap on locomotion was blocked in Q67;TU3311 worms fed with bacteria containing the *sir-2.1* RNAi plasmid as opposed to the control L4440 plasmid (Fig. 4D).

We also observed similar effects of *sir-2.1* RNAi in the single transgenic Q67-expressing worms (Fig. S2). Collectively, these data indicate that β-lap eradicated Q67 aggregates and ameliorated the associated cytotoxicity in a sir-2.1-dependent manner.

## Discussion

Many neurodegenerative diseases, such as the polyQ diseases, are associated with the formation of intracellular aggregates of mutant proteins. PolyQ diseases make up a group of inherited disorders caused by abnormally long polyQ tracts in the mutant proteins. HD is the most common of the polyQ diseases, which also include spinocerebellar ataxias, spinobulbar muscular atrophy, and dentatorubral-pallidoluysian atrophy [22]. Thus far, efficient strategies for treating experimental models of HD include 1) the delivery of small molecules or nucleic acids to target aggregate formation; 2) the degradation of mutant Htt; and 3) the elimination of protein interactions and cellular events disrupted by mutant Htt [23].

A number of previous studies showed that Sirt1 plays a protective role in neurodegenerative diseases including HD. The activation of sir-2.1 by treatment with resveratrol, a sirtuin activator, or overexpression of sir-2.1 rescued neuronal dysfunctions caused by overexpression of polyQ proteins [24] via the integrated signaling of Sirt1, β-catenin, and FOXO in C. elegans [25]. Overexpression of Sirt1 also protected neurons from mutant Htt-mediated toxicity via the deacetylation of FOXO3a, a pro-survival factor [26], or via the deacetylation of TORC1, a brain-specific modulator of CREB activity [27], in a mouse model of HD. Therefore, increasing Sirt1 activity appears to be a promising therapeutic modality for treatment of HD.

Recent studies show that degradation of disease-related mutant proteins is highly dependent on autophagy in addition to the ubiquitin-proteasome system [28], [29]. These studies suggest that autophagy can act as a cytoprotective mechanism to prevent various diseases, whereas defects in the autophagic process can lead to pathology. For example, upregulation of autophagy by inhibiting mammalian target of rapamycin (mTOR) protects against neurodegeneration in *Drosophila* and murine polyQ disease models [6]. Furthermore, chaperone-mediated autophagy contributes to the specific degradation of mutant Htt in cultured cells and in the R6/2 genetic murine model of HD [30]. Sirt1is a convincing candidate for the regulator of autophagy. The functions of Sirt1 often appear to be the primary endpoints of autophagy [11], [31]. Accordingly, Sirt1 can regulate autophagy in both *in vitro* and *in vivo* models [12]. Essential components of the autophagy machinery, such as Atg5, Atg7, and Atg8, interact with Sirt1 and are subsequently deacetylated in an NAD^+^-dependent manner, whereas the absence of Sirt1 considerably augments the acetylation level of these proteins. In addition, embryos and neonatal mice lacking Sirt1 accumulate abnormal organelles; however, transient overexpression of Sirt1 restores a basal level of autophagy [12].

Although it is widely accepted that the elimination of polyQ aggregates is an attractive therapeutic strategy for the treatment of HD, this notion has been challenged. Rather, the formation of intranuclear inclusions is considered by some authors to be a protective mechanism against toxic mutant Htt proteins [32], [33]. This is consistent with our finding that the cell death mediated by HttEx1(97Q)-GFP expression is as strong in aggregation-free cells as in aggregation-containing cells (Fig. 1B). Therefore, it is possible that autophagy is cytoprotective because it increases the elimination the toxic HttEx1(97Q)-GFP proteins, irrespective of whether they are soluble or aggregated.

β-lap is a quinone-containing compound originally isolated from the bark of the South American Lapacho tree (*Tabebuia avellanedae*) [34]. This compound has a number of pharmacological effects that are linked to the formation of reactive oxygen species, including anti-bacterial, anti-fungal, anti-trypanocidal, and cytotoxic activities [35], [36]. β-lap also shows anti-cancer activity against a number of human breast and prostate cancer cell lines [37], [38], [39]by selectively inducing NQO1-dependent apoptosis, as well as inducing NQO1-independent apoptosis in the HepG2 hepatoma cell line through the induction of Bax and the activation of caspases [40]. Although β-lap can induce death in cancer cells, the mechanism of the observed apoptosis is not well understood. NQO1 mediates the reduction of β-lap by using NADH as an electron source [13]; however, the reduced form of β-lap is unstable and is rapidly re-oxidized to the original form of β-lap. This fruitless cycle between the oxidized and reduced forms of β-lap is thought to increase the NAD^+^/NADH ratio. Because Sirt1 activity strictly requires NAD^+^ as a cofactor, β-lap is also implicated in the amplification of Sirt1 activity by elevating cellular NAD^+^ levels [14], [15].

The present study showed that β-lap decreased cell death stemming from the expression of expanded polyQ-containing Htt exon1 proteins in human neuroblastoma SH-SY5Y cells (Fig. 1A). Furthermore, β-lap reduced the formation of intracellular polyQ aggregates (Fig. 2). These actions appeared to be mediated by autophagy (Fig. 2) and β-lap-mediated activation of Sirt1 (Fig. 3). β-lap also effectively alleviated locomotion defects and reduced the number of polyQ puncta in *C. elegans* lines expressing Q67-CFP fusion proteins (Fig. 4). To the best of our knowledge, this study is the first to demonstrate that pharmacological activation of Sirt1 by β-lap is a potential strategy for the treatment of HD.

A recent study showed that oral administration of β-lap in appropriate doses prevented obesity and obesity-related metabolic phenotypes in mice [14], while another study demonstrated that β-lap prevented arterial restenosis in rats through the activation of AMP-activated protein kinase (AMPK) [15]. In light of these observations, and the dual pro-apoptotic and anti-cytotoxic actions of β-lap described above, it is certainly warranted to test the efficacy of β-lap in higher animal models of HD.

## Materials and Methods

### Cell Culture and DNA Transfection

Human neuroblastoma SH-SY5Y cells were grown in Dulbecco’s modified Eagle’s medium (Hyclone) supplemented with 10% foetal bovine serum (Hyclone) and 100 U/100 µg/ml penicillin/streptomycin (Invitrogen). Cells were transiently transfected with plasmid DNA using Lipofectamine LTX (Invitrogen) according to the manufacturer’s instructions. The pcDNA-HttEx1(25Q) and pcDNA-(97Q)-GFP constructs encoding the human Huntingtin exon1 region with 25 or 97 glutamine repeats followed by an enhanced GFP (EGFP) sequence were created as described previously [41]. A matching vector without an insert (pcDNA3, Invitrogen) was used as the empty vector control.

### Chemicals and Reagents

The anti-cancer agent β-lap was dissolved in dimethyl sulfoxide (DMSO) (Sigma) at 50 mM and further diluted to 50 µM in DMSO just before use. SH-SY5Y cells were treated at 37°C for 12 h with either DMSO (vehicle control) or the β-lap solution at a final concentration of 30 nM. The anti-autophagy agent 3-MA (Sigma) was dissolved in water at 100°C for a few minutes to yield a 100 mM stock solution. The stock solution was stored at –20°C, and 3-MA was again completely dissolved by treatment at 100°C just prior to use. Unless otherwise noted, all other chemicals and reagents were purchased from Sigma.

### Quantification of Cell Death

MTT (3-[4,5-dimethylthizaol-2-yl]-2,5-diphenyl tetrazolium bromide; Sigma) was dissolved in phosphate buffered saline (PBS) to yield a stock solution of 2.5 mg/ml. A volume of MTT solution equivalent to 20% of the culture medium volume was added to the cell culture at 37°C for 2 h. A volume of DMSO equivalent to the culture medium volume was then added, and the cell culture was placed on a shaking table until the resultant formazan crystals were completely dissolved. The absorbance of the samples was measured at 570 nm, and the background absorbance of each well was measured at 690 nm. The HttEx1(25Q)-GFP- and HttEx1(97Q)-GFP-transfected SH-SY5Y cells are differentially sensitive to oxidative stress, as assessed by exposure to H_2_O_2_ [17]. Therefore, the cytoprotective effects of β-lap were measured by the inhibition of cell death induced by 200 µM H_2_O_2_. Cell death percentages were calculated by determining the ratio of PI-stained dead cells to Hoechst 33342-stained cells (Hoechst 33342 stains the nuclei of all cells, dead or alive). To detect caspase-3 activation, cell lysates were separated for sodium dodecyl sulphate-polyacrylamide gel electrophoresis (SDS-PAGE) and probed with an antibody against the cleaved form of caspase-3.

### Western Blot Analysis and Filter Retardation Assay

SH-SY5Y cells were harvested and resuspended in RIPA lysis buffer (1% NP-40, 50 mM Tris-HCl [pH 7.4], 150 mM NaCl, and 10 mM NaF) supplemented with a mammalian cell protease inhibitor cocktail. They were then briefly sonicated as previously described [41]. The soluble protein fraction was recovered after centrifugation at 16,000×g for 10 min. Equal amounts of protein in the soluble and insoluble fractions, as determined by the bicinchoninic acid (BCA) Protein Assay (Pierce), were subjected to SDS-PAGE and transferred to a polyvinylidene difluoride (PVDF) membrane (Bio-Rad Laboratories). Membranes were blocked with 5% non-fat dry milk powder in Tris buffered saline-Tween 20 (TBS-T) for 1 h and incubated overnight at 4°C with rabbit polyclonal anti-active caspase-3 (ab13847, Abcam), rabbit polyclonal anti-LC3 (NB100-2220, Novus Biologicals), mouse monoclonal anti-glyceraldehyde 3-phosphate dehydrogenase (GAPDH) (ab9484, Abcam) or rabbit anti-Sirt1 antibodies (kindly provided by Dr. Ja-Eun Kim, Kyung Hee University). After incubation with secondary peroxidase-labelled anti-mouse or anti-rabbit antibodies (1∶10,000, Zymed Laboratories), the immune complexes were visualized using an enhanced chemiluminescence (ECL) reagent (Amersham Pharmacia). Equal protein loading was confirmed by probing for GAPDH on the same membrane, and the intensity of each band was quantified using NIH ImageJ software (available at http://rsb.info.nih.gov). The filter retardation assay was performed as described elsewhere, using the insoluble cell lysate fraction as the source of polyQ aggregates [42].

### Immunoprecipitation

SH-SY5Y cell lysates (3 mg) were mixed with anti-acetyl-lysine agarose beads (Immunechem) at 4°C overnight. Immune complexes were washed three times with lysis buffer (50 mM Tris-HCl [pH 7.4], 150 mM NaCl, 1% NP-40, 1% Sodium deoxycholate, 1% Triton X-100, 10 µM Trichostatin A, and 10 mM Nicotinamide) supplemented with a mammalian cell protease inhibitor cocktail. After boiling in 2X SDS sample buffer, equal amounts of protein were subjected to SDS-PAGE. After transfer to a PVDF membrane, the membrane was immunoblotted with rabbit polyclonal anti-PARP-1 (Cell signalling) or rabbit anit-Atg5 antibodies (kindly provided by Dr. Yong-Keun Jung, Seoul National University).

### Localization of FOXO1-GFP

The FOXO1-GFP construct was purchased from Addgene [19]. SH-SY5Y cells transfected with FOXO1-GFP were incubated in complete DMEM medium containing sirtinol or DMSO, and were then treated with β-lap for 1 h. The cells were washed three times with PBS and fixed in 4% paraformaldehyde. Images were obtained using a Fluoview FV 1000 confocal laser-scanning microscope equipped with 60X oil-immersion objectives and capable of additional 3X to 4X zoom (Olympus Optical).

### Fluorescence Microscopy and Estimation of polyQ Aggregation

SH-SY5Y cells were cultured on poly-L-lysine-coated coverslips, fixed with 4% paraformaldehyde (pH 7.4) in TBS for 10 min, and stained with Hoechst 33342. After three washes with TBS containing 1 mM CaCl_2_ and 1 mM MgCl_2_, the cells were mounted on microscope slides in PermaFluor Aqueous Mountant (Lab Vision Corporation). Fluorescence images were visualized by using a Leica DMRBE microscope equipped with a 63×(1.4NA) oil immersion objective and fluorescein isothiocyanate- or Texas Red-optimized filter sets (Omega^R^ Optical Inc). Images were acquired with a CoolSNAP ™ fx CCD camera (Photometrics) and analysed with Metamorph imaging software (Universal Imaging Co). To determine the levels of polyQ aggregation among GFP-positive cells, the number of aggregation-positive cells relative to the total number of GFP-positive cells was counted in 40 random fields per culture.

### RNA Silencing with shRNAs

Scrambled and Sirt1 shRNAs were designed by GenSKript. The sequences of the shRNAs were as follows: sh-Scr, GTGAA GAGAA AGGAG TCGA ATCTT GATAT CCGGA TTCGA CTCCT TCTCT TCACT TTTTT CCAA; sh-Sirt1, AAGTT ACTGC AGGAG TGTAA ATTGA TATCC GTTTA CACTC CTGCA GTAAC TTTTT TTTCC AA. The shRNAs were subcloned into the RNAi-Ready pSIREN-DNR-DsRed-Express Donor Vector (Clontech) via BamHI and EcoRI sites. The resulting vectors were transfected into SH-SY5Y cells. After incubation for 36 h, the silencing effect of the shRNAs was examined by Western blotting.

### *C. elegans* Strains and RNAi Feeding Protocol

The following transgenic stable worm lines were obtained from the Caenorhabditis Genetics Center (CGC, College of Biological Sciences, University of Minnesota): (19Q) AM49 rmIs172 [F25B3.3p::Q19::CFP], (67Q) AM44 rmIs190 [F25B3.3p::Q67::CFP], and TU3311 (*Punc-119*::*sid-1*) [20], [43]. Nematodes were grown and maintained on nematode growth medium (NGM)-agar plates using standard methods [44]. RNAi was performed essentially as previously described [45]. Briefly, bacteria containing the *sir-2.1* RNAi plasmid (MRC Geneservice) or empty vector L4440 were cultured in LB medium containing 100 µg/ml ampicillin for 6–18 h at 37°C. The cultured bacteria were then seeded onto RNAi plates (NGM-agar containing ampicillin (100 µg/ml) and 1 mM isopropylthio-β-galactoside (IPTG)) and induced overnight at room temperature. L4 stage worms of the F2 generation were transferred into M9 media containing 25 µM β-lap and 1.25% DMSO and then aliquotted onto the RNAi plates. Twenty four hours later, the locomotion rate was measured by counting body bends (defined as when the part of the worm behind the pharynx reaches a maximum bend in the opposite direction from the previous bend) for 1 min at 20°C. Alternatively, worms were paralyzed on agarose pads using 5 mM sodium azide, and images of polyQ-derived fluorescence were captured by using a Leica DMRBE microscope equipped with a CoolSNAP ™ fx CCD camera and analysed with Metamorph imaging software (Universal Imaging Co). The number of polyQ aggregates per worm was counted.

### Statistical Analysis

Results are expressed as the mean ± SDs. Comparisons between two groups were performed using the Student’s *t*-test. Comparisons between multiple groups were made by one-way ANOVA with the Bonferroni correction. Statistical analyses were conducted with Statview software version 5.0 (SAS Institute Inc.). A *p*-value of less than 0.05 was considered statistically significant.

## Supporting Information

Figure S1 **Knockdown of Sirt1 expression**. To examine the effects of sh-Sirt1 on the expression level of Sirt1, Western blotting was performed. Human neuroblastoma SH-SY5Y cells were transfected with a plasmid harbouring scrambled shRNA (sh-Scr) or Sirt1 shRNA (sh-Sirt1). Cell lysates were separated by SDS-PAGE, transferred onto a PVDF membrane, and probed with an anti-Sirt1 antibody. GAPDH (loading control) was detected with an anti-GAPDH antibody. Significant down-regulation of the Sirt1 level was observed with sh-Sirt1.
(EPS)

Figure S2 **β-lap protects polyQ-expressing neurons from toxicity in Q67-expressing worms**. The experiments shown in Fig. 4B and D were also carried out in the Q67-expressing worms and similar results were obtained. Of note, the effects of *sir-2.1* RNAi on body bending were more evident in the double transgenic Q67;TU3311 worms compared to the single transgenic Q67 worms. This observation may reflect the enhanced uptake of dsRNA into neurons in Q67;TU331 worms. *sir-2.1* RNAi in Q67-expressing worms were performed in the presence (b and d) or absence (a and c) of 25 µM β-lap. The average number of polyQ puncta (A) and body bends during 1 min of continuous movement on agar plates (B) were quantified and plotted. Each bar and error bar represents the mean ± SD (A: n = 10, C: n = 36); *p<0.05, **p<0.01 (Student’s *t* test).
(EPS)
